# The intelligent modelling and optimization of an economic and ecosystem-friendly model for grid connected prosumer community

**DOI:** 10.1371/journal.pone.0276510

**Published:** 2023-01-20

**Authors:** Usman Mussadiq, Saeed Ahmed, Muhammad Sajid, Dalia H. Elkamchouch, Lal Hussain, Abdulbaset Gaddah, Fahd N. Al-Wesabi, Anwer Mustafa Hilal

**Affiliations:** 1 Department of Electrical Engineering, Mirpur University of Science and Technology, Mirpur (AJK), Pakistan; 2 Department of Electronics Engineering, Korea Polytechnic University, Siheung, South Korea; 3 Department of Information Technology, College of Computer and Information Sciences, Princess Nourah Bint Abdulrahman University, Riyadh, Saudi Arabia; 4 Department of Computer Science and Information Technology, King Abdullah Campus Chatter Kalas, University of Azad Jammu and Kashmir, Muzaffarabad, Azad Kashmir, Pakistan; 5 Department of Computer Science and Information Technology, Neelum Campus, University of Azad Jammu and Kashmir, Athmuqam, Azad Kashmir, Pakistan; 6 Department of Computer Sciences, College of Computing and Information System, Umm Al-Qura University, Mecca, Saudi Arabia; 7 Department of Computer Science, College of Science & Art at Mahayil, King Khalid University, Abha, Saudi Arabia; 8 Department of Computer and Self Development, Preparatory Year Deanship, Prince Sattam Bin Abdulaziz University, AlKharj, Saudi Arabia; Wuhan University, CHINA

## Abstract

The establishment of grid-connected prosumer communities to bridge the demand-supply gap in developing nations, especially in rural areas will assist to minimize the use of carbon enriched fossil fuels and the resulting economic pressure. In the promoted study, an economic and ecosystem-friendly hybrid energy model is proposed for grid-connected prosumer community of 147 houses in district Kotli, AJK. The grid search algorithm-based HOMER software is used to simulate and analyze the load demand and biomass sources-based onsite collected data through a survey for an optimal proposed design. The research objectives are to minimize the net present cost (USD) of design, the per unit cost of energy (USD/kWh), and the carbon emissions (kgs/year). A sensitivity analysis based on photovoltaic module lifetime is also performed. The simulations show that the per unit cost of energy is reduced from 0.1 USD/kWh to 0.001 USD/kWh for the annual energy demand (kWh/year) of the community. The number of carbon emissions is also minimized from 122056 kgs/year to 1628 kgs/year through the proposed optimal energy model.

## 1. Introduction

In the next decade [1], the world population can rise to 50% resulting in a 25% increase in the demand-supply gap. According to a recent global survey [2], energy usage will rise to 28% between 2015 to 2040. Uninterrupted electrical power has played a vital role in the development of progressing nations [3, 4]. Currently, 1.2 billion people across the globe and 25 million [5] people in Pakistan are deprived of direct energy access. The reasons behind these energy crises are population enlargement, energy losses due to inefficient distribution systems, economic constraints, varying energy policies and natural calamities [6]. The excessive use of fossil fuels to meet such high energy demands is the cause of 89% of carbon emissions [7]. Fossil fuels-based electrical energy generation contributes 40% of these emissions [8]. The carbon-enriched sources are transformed into carbon-free renewable resources for energy production across the globe [9]. However, only 27% individuals are convinced to use these ecosystem-friendly resources [10]. Among developing nations, Pakistan is importing carbon-enriched energy sources to meet her energy demand by spending 20% of economy. The use of crude oil, ultimately results in the greenhouse effect coupled with multiple health issues [11]. The government of Pakistan has planned to increase the renewable energy share from 1% to 5% by 2030 [12]. About 140 million individuals are facing 10–12 hours of undue load shedding in rural areas [13]. Among various solutions, the load curtailments can be reduced by the actively participation of end users to generate energy for their own needs by exploiting available renewable energy resources. These active users are named prosumers who can produce and consume energy simultaneously. The participation of these users can be improved by using various motivational models [14] to improve energy reliability and social acceptance which in turn reduce the per unit cost of energy (COE) and number of carbon emissions [15]. The surplus power of these users is shared with the grid or nearby community by using different trading systems i.e., net metering [16] and peer-to-peer [17]. The traditional grids need to be transformed into smart grids for handling surplus power flow [18] which makes the power system operations more complex due to the non-linear and uncertain power supply of these energy resources [19]. The flexible load i.e., electric vehicles, air conditioners, and energy storage systems also increases the power systems’ operational complexity [20]. However, the grid transformation results in various issues related to data collection, data monitoring, data transmission, data privacy, sensing infrastructures, demand-supply response and energy efficiency [21]. The use of renewable energy resources at users’ ends result in microgrids which may be grid-connected or isolated hybrid energy systems (HES). The composition of HES is based on site location [22], community load profile, RER potential, power quality and investment capacity [23]. In technical literature, a variety of research studies have been conducted to check the economic feasibility of grid-connected and isolated microgrids for electrification by using different optimization techniques globally [24].

For instance, the authors have analyzed the economic feasibility of PV-wind turbine systems along with different storage systems in Australia. The study has proved that the hydrogen cell-based energy storage system is more economical than the conventional storage system [25]. A PV-wind turbine-fuel cell HES is proposed through HOMER simulations in Malaysia [26]. In [27], the authors did a Simulink-based comparative analysis between wind energy systems and hydrokinetics to check the economic feasibility. The authors concluded that a permanent magnet synchronous generator (PMSG)-based hydrokinetics system is more economical than wind energy systems. The PV modules are used as an isolated grid for a DC supply to solar homes in Africa [28]. In Uganda, an off-grid photovoltaic system is simulated by using MATLAB to propose an economic energy system [29]. A PV-hydro-diesel generator based HES is proposed in Iraq by using HOMER software [30]. In [31], the authors have used particle swarm optimization in MATLAB to propose the optimal HES which consists of PV and battery storage system along with standby diesel generator to minimize the load shedding in Quetta, Pakistan. The optimal size of PV modules, wind turbines, and battery storage systems along with diesel generators as a backup power source is proposed by using particle swarm optimization to minimize per unit of cost of energy in Saudi Arabia [32]. In [33], the authors have used the decision-making algorithms on ten different HES for sustainable power supply with less carbon emissions in Ghana. Among the ten different combinations of HES, the photovoltaic-wind-storage batteries-natural gas system is the most optimal system. Similarly, another study in Ghana is carried out to estimate the economic feasibility of different HES through HOMER simulations [34, 35]. In [36], the authors have used multi-objective particle swarm optimization (MOPSO) and multi-objective crow search (MOCS) algorithms to propose the optimal design of HES for economic and reliable power supply. The result has concluded that MOPSO is better than MOCS. The list of abbreviations is presented in Table 1. Table 2 presents a brief analysis of existing studies and the research gap.

**Table 1:** List of abbreviations.

Abbreviation	Description
NASA	National aeronautics and space administration
HOMER	Hybrid optimization of multiple electric renewables
NREL	National renewable energy laboratory
HEM	Hybrid energy model
HES	Hybrid energy system
PMSG	Permanent magnet synchronous generator
MOPSO	Multi-objective particle swarm optimization
MOCS	Multi-objective crow search
COE	Cost of Energy
NPC	Net present cost
USD	United state dollars
O&M	Operation and maintenance cost

**Table 2:** Related work.

References	Energy sources	COE optimization	Simulation’s tool	CO_2_ emissions estimation	Surplus power calculation
[27]	Wind, hydrokinetics	Yes	MATLAB	No	No
[28]	PV, Grid	No	MATLAB	No	No
[29]	PV	Yes	MATLAB	No	No
[30]	Hydro, PV, Diesel	Yes	HOMER	No	No
[37]	PV, Wind, Biogas	Yes	HOMER	No	No
[38]	PV, Biogas	Yes	HOMER	No	No
Proposed	PV, Biogas, Grid	Yes	HOMER	Yes	Yes

Keeping in view the above-mentioned studies, it can be concluded that the existing works lack the following:

- No study is reported for the region of Azad Jammu and Kashmir to promote the integration of HES at the users’ end for a sustainable power supply economically with less carbon emissions to develop the grid-connected prosumer communities.
- A detailed analysis by using HOMER simulations to estimate the surplus power share, amount of carbon emissions, the per unit cost of energy, and the net present cost is missing to the best of the author’s knowledge.
- Sensitivity analysis-based on the lifetime of PV modules to estimate the impact on the per unit cost of energy (USD/kWh), the net present cost (USD) and optimal capacity (kW) are also missing.

The rest of the paper is organized as follows. Section 2 is dedicated to load and resource estimation. The system modelling and objective function is presented in Section 3. Section 4 is dedicated to the optimization tool. The results are analyzed and described in Section 5 to propose an optimal HEM for grid-connected prosumer community. Finally, the conclusions are drawn.

## 2. Load and resource estimation

The proposed idea is aimed to present an affordable gap bridging solution to the demand-supply disparity of a small community facing the problem of electric energy shortage. To model the problem, a community, consisting of 147 houses in a remote village of District Kotli that is facing frequent unavailability from the AC power supply, is selected. The data is collected through an onsite quantitative survey conducted over a sample (prosumer community). The verbal consent of the participants of the survey is sought by knocking doors. Each participant (house owner) is contacted and briefed about the importance of study and its impact on their electricity supply and consumption. Once the participant is satisfied, the survey questionnaire is distributed, and data collection of the subject house/ sample is carried out. The survey is conducted as follows:

- The electric utility bill of each house is collected to determine total number of consumed units (kWh) in a month.
- The per day average consumption of the electrical energy, *E*_*d*,*avg*_, is calculated from the monthly electricity consumption of all samples, *E*_1_ + *E*_2_ + ……. + *E*_147_, in kWh by using Eq 1.
$$E_{d,avg}=\frac{\left(E_{1}+E_{2}+E_{3}+\ldots \ldots +E_{147}\right)}{30}.$$ (1)
- The participants are asked to provide information about their biomass sources as given:
NumbersofCowsBuffalosheifersPoultrybirdsYoungCattlesXXXXXXXXXX

HOMER is used to model three types of loads i.e., primary load, deferrable load, and thermal load. TV, radio, lighting, computers, home appliances are modeled as primary load for prosumer community. Solar, wind, hydro and biomass are used to develop a hybrid energy model in HOMER. Among these resources, the solar and biomass resources are available at targeted site and used in this paper.

### 2.1 Ethical statement

Not Applicable.

### 2.2 Community load profile

**Fig 1:**
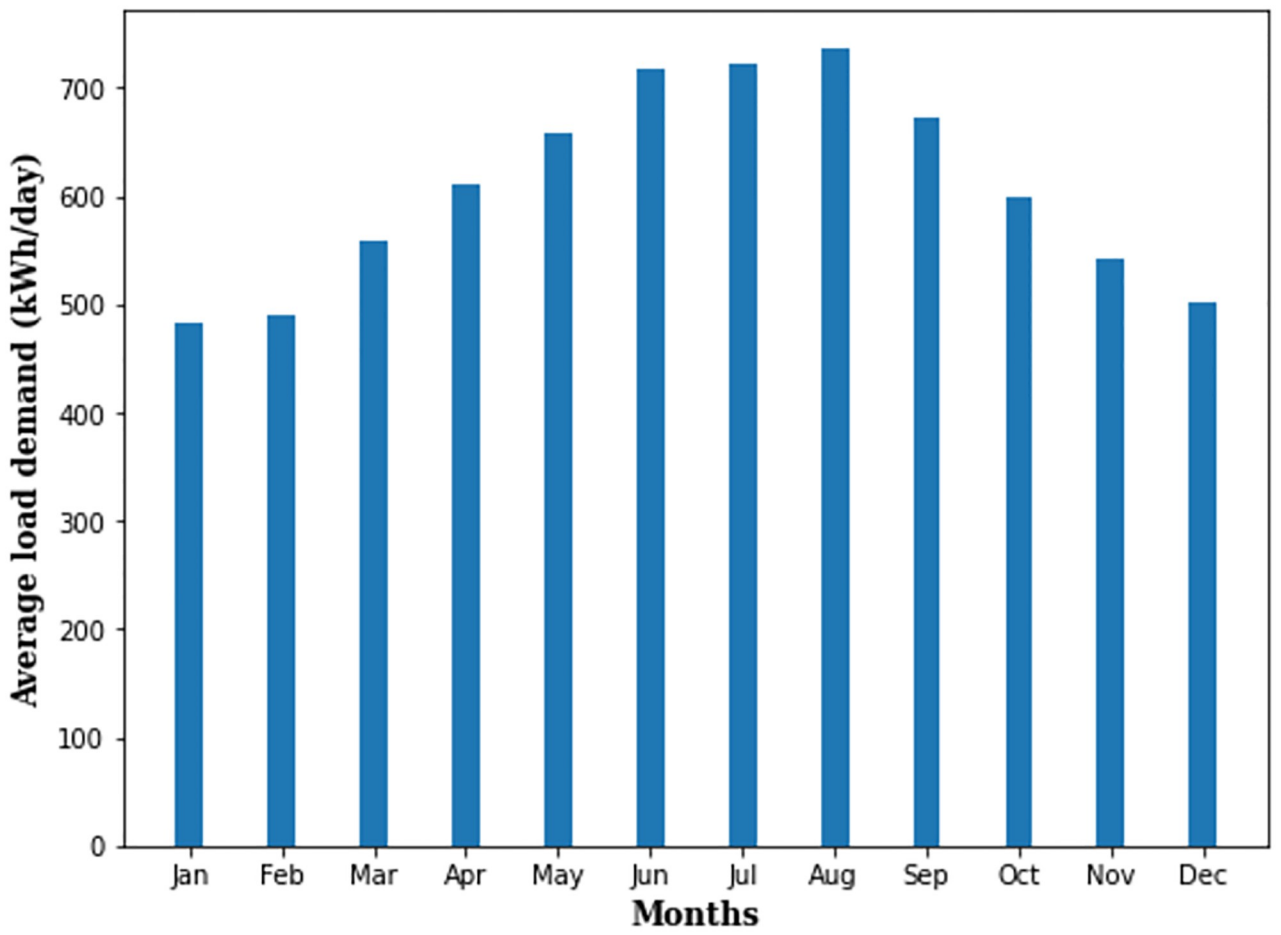
The community load profile.

The community load profile is shown in Fig 1. The monthly average load and peak demand of the community is 608 kWh/day and 85.60 kW with 30% load factor, respectively. It is clear from Fig 1 that the monthly average load is not uniform throughout the year. For instance, the average load is 481.68 kWh/day in January and 735.84 kWh/day in August.

### 2.3 Solar radiations

**Fig 2:**
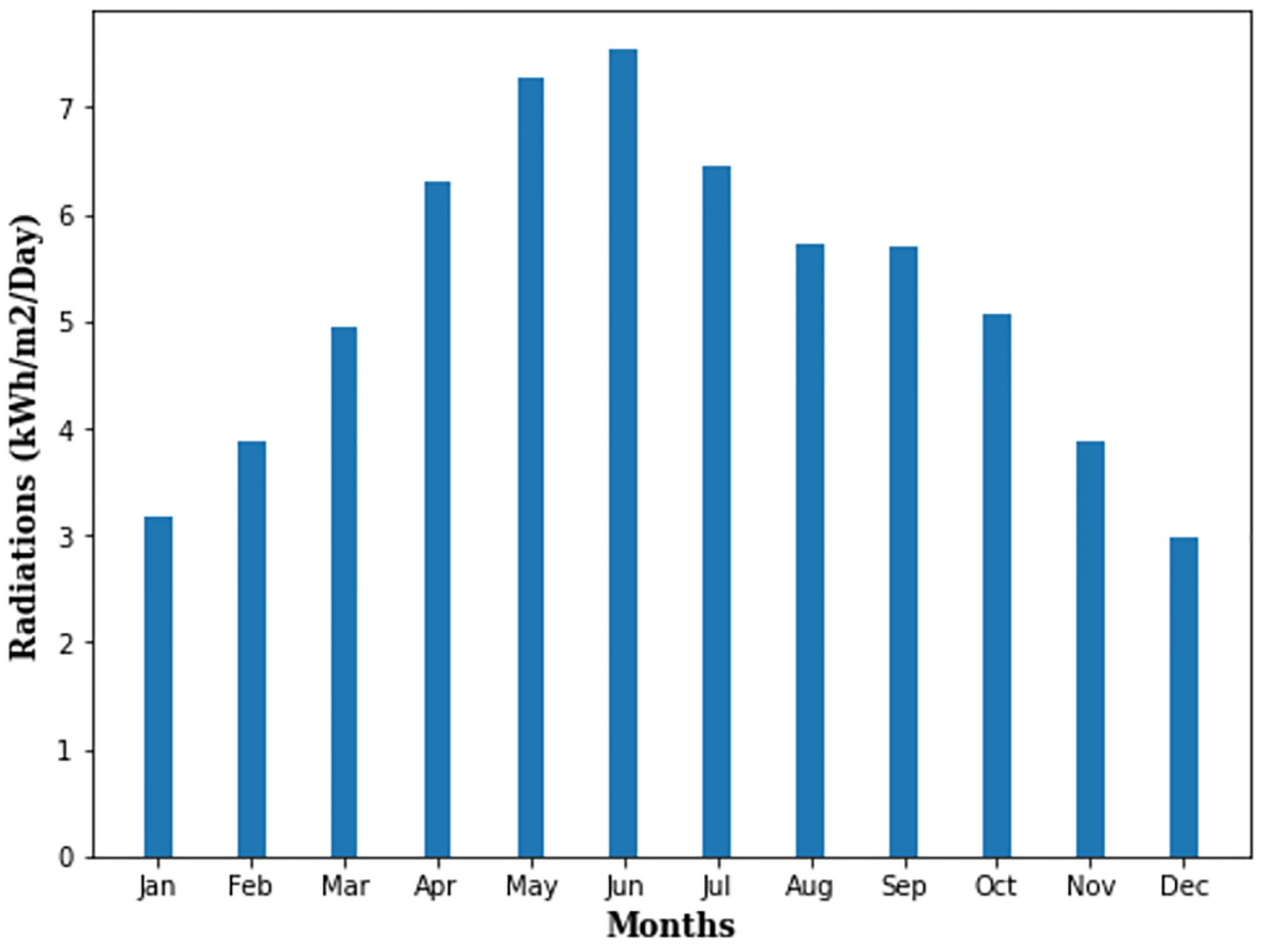
The solar radiations at targeted site.

The solar radiations-based data is collected through the NASA website for the prosumer community which is located at the longitude 33.5008° N and 73.9007° E. Fig 2 shows the average monthly radiations (kWh/m^2^/day) at targeted site.

The average monthly radiation potential is also not uniform throughout the year for prosumer community. For example, the targeted site receives 7.54 kWh/m^2^/day of radiations in June while in December, only 2.99 kWh/m^2^/day of radiations are available for energy generation.

### 2.4 Biomass potential

The biomass sources of the community are estimated through a survey. Table 3 presents that the total biomass potential of the community is 1660 tons/year. The biomass potential of the available biomass sources is calculated by using Eqs 3 [39] and 4 [39] which are presented in the subsequent section. This is another source of clean energy at targeted site.

**Table 3:** Community biomass potential.

Biomass sources	Number of these animals	Biomass potential (Tons/year)
Buffalo	87	640
Cows	53	458
Heifers	37	296
Young Cattle	22	244
Poultry	1000	22

The monthly average biomass potential is 4.54 (Tons/day) of the community. The biomass potential is uniform throughout the year and the seasonal variations have no impact on this source of green energy. HOMER calculates the amount of electrical energy directly from the biomass potential (Tons/day).

## 3. System modelling and objective function

In this section, the description of different hybrid energy models is added. The mathematical and economical modelling for system components is also added along the objective function.

### 3.1 Model description

Three different hybrid energy models (HEM) are proposed based on available energy resources for the electrification of grid-connected prosumer community as shown in Table 4.

**Table 4:** Various possible hybrid energy models.

HEM	HEM-A	HEM-B	HEM-C
Description	PV-grid	Biomass-grid	PV-biomass-grid

Model-A is on grid photovoltaic system. PV systems are source of green energy and grid is used as a backup power source in absence of enough solar potential. PV systems may be replaced by adding biomass source of energy due to its uniform availability in model-B. However, in model-C, all the available sources are used together. PV modules, bi-directional system converters, and biomass-based genset are major system components which are used to develop these models. The mathematical modelling and economic feasibility of these components is presented hereby.

### 3.2 PV modules

The solar radiations incident at photovoltaic modules and then transformed into electrical energy. The output power of PV systems is calculated by using Eq 2 [40] in HOMER.


$$P_{pv}=y_{{}_{pv}}f_{pv}\left(\frac{G_{T}}{G_{T,STC}}\right).$$ (2)

Where, *y*_*pv*_ and *f*_*pv*_ denotes the rated capacity of PV module (kW) and its derating factor, respectively. The global solar radiations (kWh/m^2^) incident at operating temperature and standard testing conditions on PV module are denoted by *G*_*T*_ and *G*_*T*,*STC*_, respectively. The effect of voltage and temperature is not considered. The derating factor estimates the effect of dust, wire losses, and environment temperature on the rated output power of the module. The generic flat plate PV modules having 13% efficiency and lifetime of 25 years is used for simulations. The modules’ ground reflectance is 20% and derating factor is 80%, respectively. The initial capital cost of PV module is 995 USD/kW [40]. The replacement cost is 796 USD/kW which is equal to the 80% of initial capital cost. The PV modules are fixed and need cleanliness only twice in a month for generating electricity at rated efficiency. Hence, 2 USD/kW is considered as operation and maintenance cost.

### 3.3 Biomass-based genset

The biomass-based generator-set is used to generate the electrical energy for the prosumer community. The total biomass potential which is calculated from all the available sources i.e., cows, buffaloes, heifers, and poultry is transformed into heat energy, *E*_*b*_. The heat energy is used to generate electricity, *P*_*bg*_. Eq 3 [39] is used to estimate the biomass waste, *m*(tons/year), of cows and buffaloes having milk production *Y*_*d*_(kg/year).


$$m=0.0024Y_{d}+0.447.$$ (3)

Eq 4 [39] is used to estimate the biomass waste of heifers, poultry birds, and young cattle. Here *N*_*i*_ represents the average number of livestock within a specified region and *m*_*i*_ shows the manure produced in a year. The value of *m*_*i*_ [39] for poultry birds, heifers, and young cattle is 0.022, 8, and 11.1, respectively.


$$M=\sum_{n=1}^{i}N_{i}m_{i}.$$ (4)

The volume of biogas, *V*_*b*_(m^3^), from the total livestock waste in a year is calculated by using Eq 5 [39]. *N* represents the total number of animals in *i*^*th*^ group.


$$V_{b}=\sum_{n=1}^{i}N_{i}m_{i}k_{DM,i}k_{OM,i}v_{b,i}.$$ (5)

*k*_*DM*,*i*_ and *k*_*OM*,*i*_ is the amount of dry matter content in waste and organic content in dry matter, respectively. *v*_*b*,*i*_ is the definite output of biogas (m^3^/ton) from *i*^*th*^ group of animals. The definite heat energy, *E*_*b*_ (kWh), is calculated from the Eq 6 [41].


$$E_{b}=\sum_{n=1}^{i}N_{i}m_{i}k_{DM,i}k_{OM,i}v_{b,i}e_{b,i}.$$ (6)

*e*_*b*,*i*_ is the specific heat energy (kWh/m^3^) which is produced by dung. This heat energy is transformed into electrical energy, *P*_*bg*_ (watts). The electrical energy is calculated in HOMER by using Eq (7) [41].


$$P_{bg}=\frac{E_{b}}{k_{e}T_{o}}.$$ (7)

Here, *k*_*e*_, the electric efficiency coefficient and it has a constant value of 0.4 during the generator operating hours, *T*_*o*_. The generator set having 25% efficiency and lifetime of 15000 hours is used for simulations. The lower heating value of the genset is 5.5 MJ/kg which is provided by HOMER. The initial capital cost is 40000 USD. The replacement cost is 32000 USD which is equal to the 80% of initial capital cost. The operation and maintenance costs are 2 USD/hour [42].

### 3.4 Bi-directional converter

The system converter is used for rectification and inversion, respectively. In rectification, the AC power is transformed into DC while in inversion, the DC power is changed into AC [43]. The initial capital and replacement costs of system converter are 600 USD/kW and 480 USD/kWh, respectively. The operation and maintenance cost is 3 USD/year [44]. The system converter is 95% efficient with the life-span of 15 years. Table 5 presents the input data for the major components of hybrid energy model.

**Table 5:** Major system component.

Component	Initial capital cost (USD/kW)	Replacement cost (USD/kW)	O&M cost (USD/year)	Lifetime (Years)
Generic flat plate PV	995	796	10	25
Generic fixed capacity genset	400	320	2 $/hour	18
Converter	600	480	3	15

### 3.5 Objective function

The research objectives are to minimize the net present cost, the cost of energy and the number of carbon emissions to propose an economic and ecosystem-friendly model for grid-connected prosumer community. The objective function is denoted by Eq 8.


$$\text{min}\left(C_{NPC}+COE+CE\right).$$ (8)

#### 3.5.1 Net present cost (USD)

The net present cost of proposed model is estimated from the annualized cost of each system component and its capital recovery factor. The net present cost is the sum of initial capital cost, replacement cost, operation and maintenance cost, salvage value and revenue from net grid sales. Eqs 9 [45] and 10 [45] is used by HOMER to calculate the annualized cost and capital recovery factor.


$$C_{an}=\delta (i,L).C_{NPC}.$$ (9)

$$\delta (i,L)=\frac{i{\left(1+i\right)}^{L}}{{\left(1+i\right)}^{L}-1}.$$ (10)

Here, *C*_*NPC*_ and *C*_*an*_ is the net present cost and annualized cost of the system respectively. *L* denotes the project lifetime, and *δ* is used for capital recovery factor. *i* denotes the annual real discount rate. The salvage value for each component is calculated by using Eq 11 [46]. *L*_*rem*_ and *L*_*comp*_ is the remaining and total lifetime of each component at the end of project. *C*_*rep*_ is the component replacement cost to estimate the salvage value, *S*.


$$S=C_{rep}*\frac{L_{rem}}{L_{comp}}.$$ (11)

#### 3.5.2 Cost of energy

The cost of energy, *COE* (USD/kWh) is calculated by using Eq 12 [47] in HOMER.


$$COE=\frac{C_{an}}{E_{load}+E_{sales}}.$$ (12)

Here, *E*_*load*_ and *E*_*sales*_ is the amount of total energy supplied to load in a year (kWh/year) and supplied to grid (kWh/year) by community, respectively.

#### 3.5.3 Carbon emissions

HOMER estimate six types of pollutants which are emitted into air. The sources of these emissions are fossil fuels-based generator, boiler, and the grid. The net grid purchases, *E*_*purchases*_ (kWh) are multiplied with the emission factor, *λ* (g/kWh) to calculate the number of carbon emissions as shown by Eq 13 [47]. The value of emission factor is 0.55kg/kWh in this paper [14].


$$CE=\lambda E_{purchases}.$$ (13)

## 4. The optimization tool

There are various optimization techniques i.e., particle swarm optimization, bat algorithms, cuckoo search algorithm, etc. which can be employed in MATLAB to propose an optimal design. However, the HOMER software is used in this research paper to propose the hybrid energy model for a targeted community on the following grounds: 1) HOMER is used across the globe for on-grid or isolated microgrids designs, 2) various recent research studies are based on HOMER simulations for an optimal economic design [43], 3) HOMER employs the updated grid search algorithm and optimizer to find the optimal configuration, and 4) HOMER easily manage the uncertainty of non-dispatchable renewable supply, variable load, and fuel prices. The authors intend to include more optimization techniques by employing MATLAB and Python in future works. HOMER assist to develop HEM through simulations [37], optimization [48], and sensitivity analysis [38]. In simulations, the different proposed hybrid energy models having a range of components’ size are simulated one by one to estimate the net present cost of each model for the targeted community. The net present cost is the sum of capital cost, replacement cost, operation and maintenance cost, the salvage and the revenues which is equal to grid sales (USD/kWh). At optimization stage, an optimal model is obtained which fulfill the users’ energy demand at minimum net present cost. The grid search algorithm simulate all models having different component sizes which are entered in search space. The components’ sizes are used as decision variables by designer. The decision variables are also named as optimization variables. The fuel price, PV lifetime, load fluctuations, and solar radiations are considered as uncertainty parameters and their impact is estimated by sensitivity analysis.

**Fig 3:**
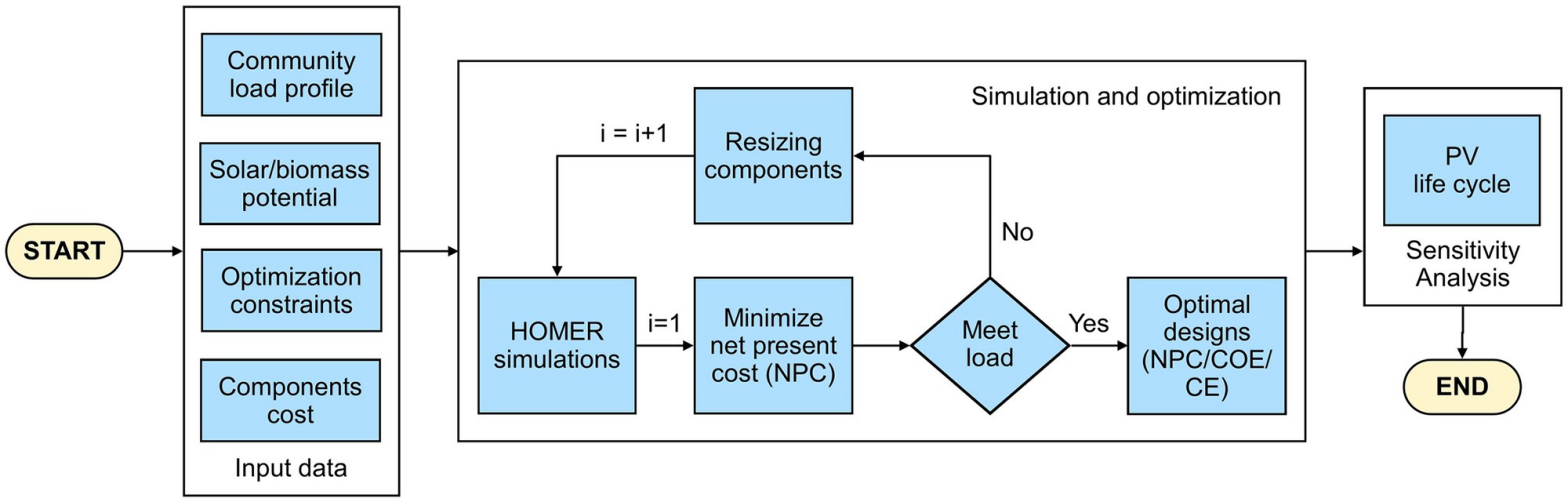
The flowchart of optimization tool.

Fig 3 shows the flowchart of the optimization process. The model inputs are community load, the solar and biomass potential, the optimization variables, and the components’ cost. The optimization variables in search space are as follows: the size of PV modules have a range of 0–900 kW, the system converter size is varied from 0–500 kW, the biomass-genset has a range of 0–100 kW, and the project lifetime is 25 years. The flowchart consists of following steps:

1. The data collection.
2. The optimization constraints.
3. The costs of major system components are used.
4. The simulations are performed for each HEM to meet the load at minimum the NPC.
5. The optimal model among proposed HEMs having minimum NPC is selected.
6. The sensitivity analysis is used to estimate the effect of PV modules’ life-span on the NPC.

## 5. Result analysis and discussion

The simulation results are analyzed in this section to propose an optimal hybrid energy model for the grid-connected prosumer community. The model is proposed for a sustainable energy supply to avoid the frequent grid supply interruptions, huge carbon emissions, and high per unit cost of 0.1 (USD/kWh). The prosumer community imports 221920 kWh/year from the grid to meet the energy demand and nearly 122056 kgs/year of carbon is emitted by the grid to generate it.

### 5.1 Hybrid energy model-A

**Fig 4:**
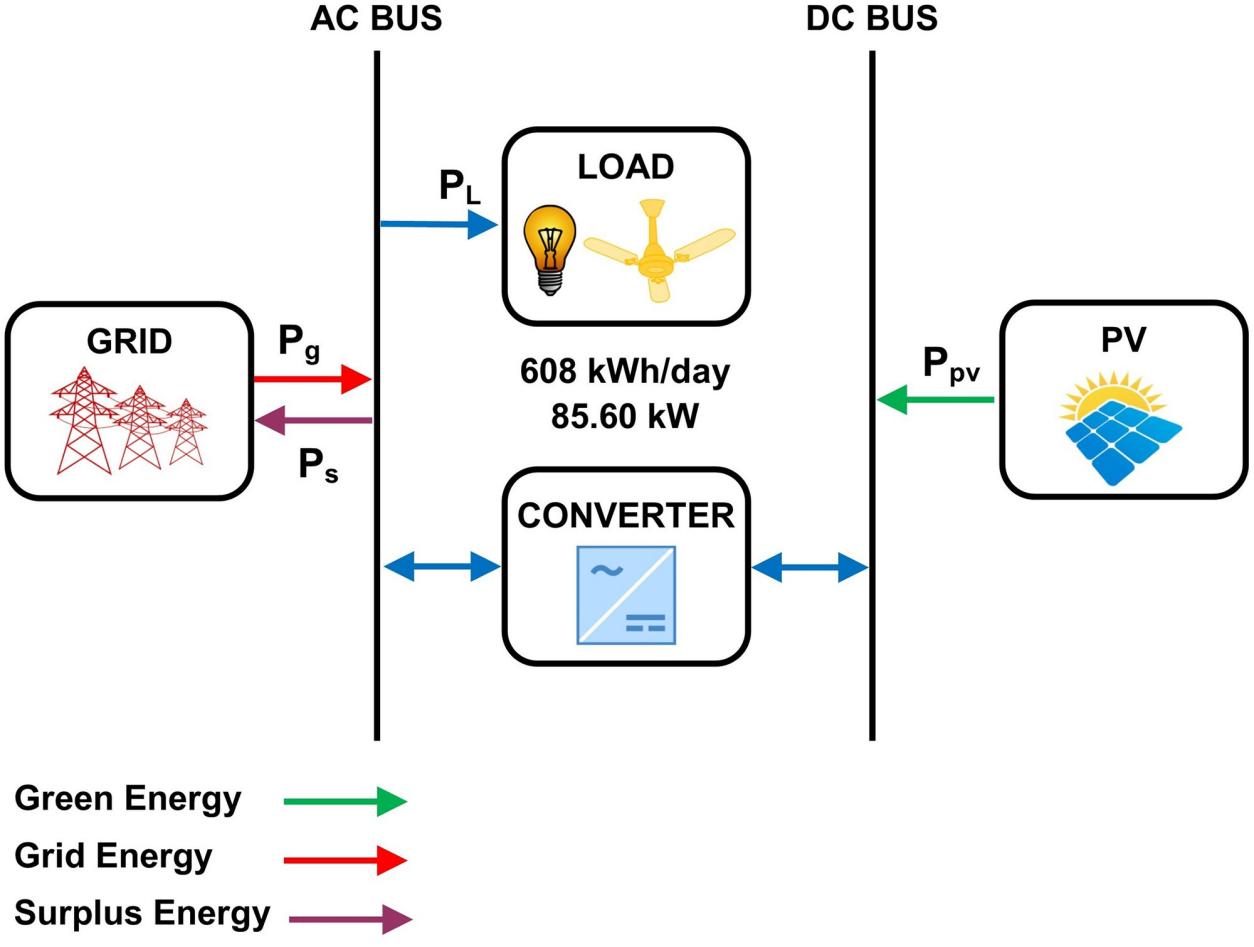
The hybrid energy model-A for the prosumer community.

In this model, PV modules, and converters are used as a system component to supply 608 kWh/day of energy for the grid-connected community. HOMER simulates 30 configurations for a search space range based on components size. Among these configurations, the optimal model has a 750 kW PV module size with capacity factor of 20%. The optimal size of the system converter is 500kW having a capacity factor of 27.3%. The conversion losses are 62967 kWh/year for the operation of 4386 hours. Fig 4 shows the hybrid energy model-A for the prosumer community.

The simulation results for HEM-A are presented in Table 6.

**Table 6:** Simulation results for HEM-A.

Component	Generation (kWh/year)	Grid sales (kWh/year)	CO_2_ emissions (kgs/year)	NPC (USD)	COE (USD/kWh)
PV	1311174 (92.8%)	1076721	55893	179732	0.01
Grid supply	101625 (7.19%)				
Total	1412799 (100%)				

It is very clear from the tabular data that the PV-based renewable energy contribution is 92.8% which is much higher than the grid energy contribution of 7.19%. The community shares 1076071 kWh/year of surplus energy with the grid at the rate of 0.08 USD/kWh which is lower than the grid energy price of 0.1 USD/kWh. A huge number of carbon emissions i.e., 55893 kgs/year is released into the surroundings due to grid energy contribution. The net present cost is 179732 USD while the per unit cost of energy is 0.01 USD/kWh which is charged by the prosumer community. The net present cost consists of capital cost, replacement cost, and operation and maintenance cost. The capital cost, replacement cost, and operation and maintenance cost are 1046250 USD, 114825 USD, and -949179 USD, respectively. A revenue of 86085 USD/year is generated by sharing surplus power to the grid and it is adjusted in NPC.

### 5.2 Hybrid energy model-B

**Fig 5:**
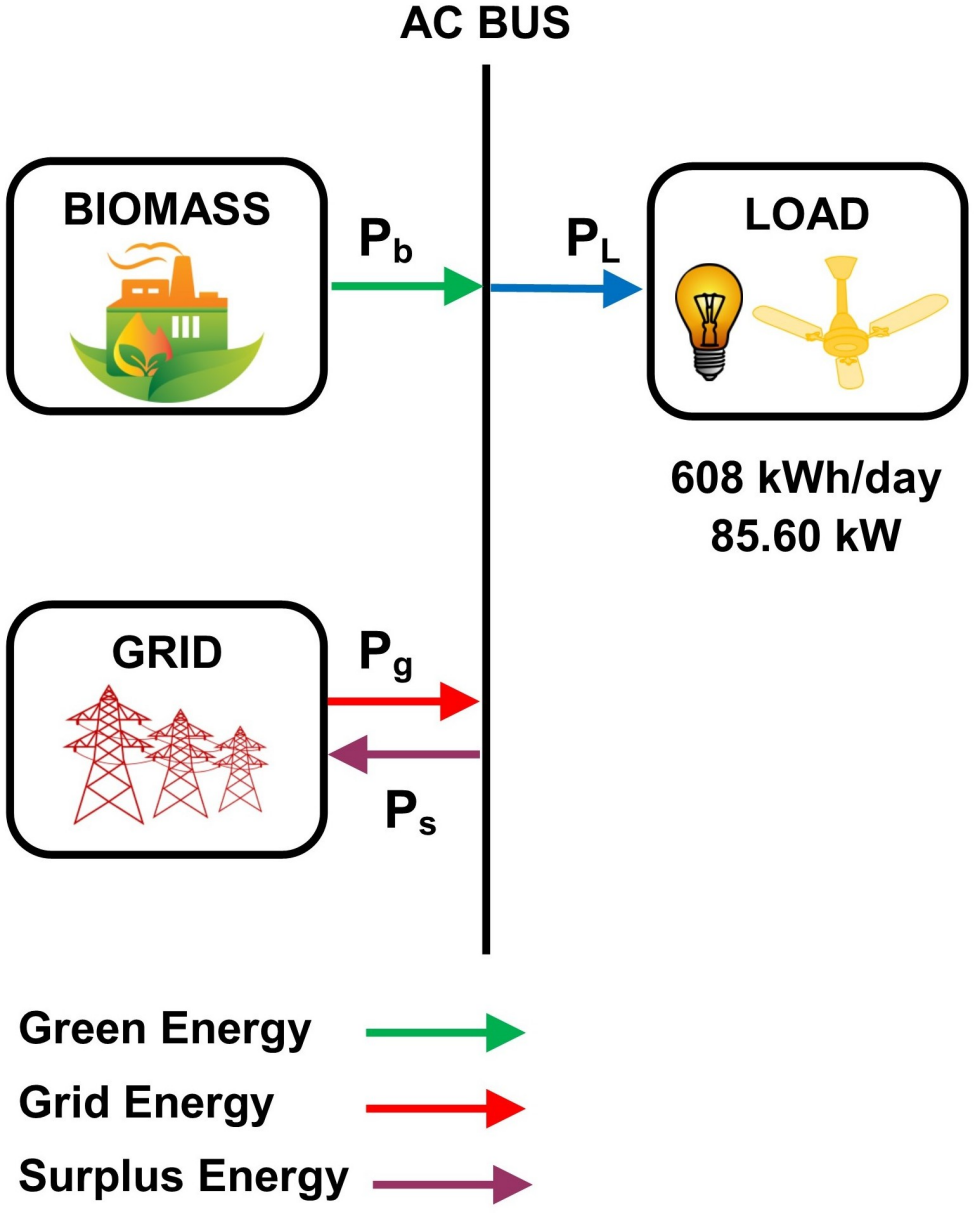
The hybrid energy model-B for the prosumer community.

In this model, the biomass-based generator is used as a system component to meet the energy demand of the grid-connected community. HOMER simulates 4 different configurations for a search space range-based on components size. Among these configurations, the optimal model has 50 kW generator size with 8760 hours of operation. The fixed generation cost is 2.07 USD/hour with 25.9% electrical output efficiency. Fig 5 shows the hybrid energy model-B for the prosumer community.

Table 7 presents the simulation results for HEM-B. The energy contribution of the biomass system is 99.2% which is much higher than the grid energy contribution of 0.8%.

**Table 7:** Simulation results for HEM-B.

Component	Generation (kWh/year)	Grid sales (kWh/year)	CO_2_ emissions (kgs/year)	NPC (USD)	COE (USD/kWh)
Biomass	438000 (99.2%)	219486	1873	25197	0.004
Grid supply	3406 (0.8%)				
Total	441406 (100%)				

The community shares 219486 kWh/year of surplus energy with the grid at the rate of 0.08 USD/kWh. The number of carbon emissions that are released into the surroundings due to grid energy contribution is 1873 kgs/year. The net present cost is 25197 USD while the per unit cost of energy is 0.004 USD/kWh which is charged by the prosumer community. The capital cost, replacement cost, and operation and maintenance cost are 20000 USD, 116074 USD, and -109344 USD, respectively. The community generates revenue of 17558 USD/year by sharing surplus power with grid.

### 5.3 Hybrid energy model-C

**Fig 6:**
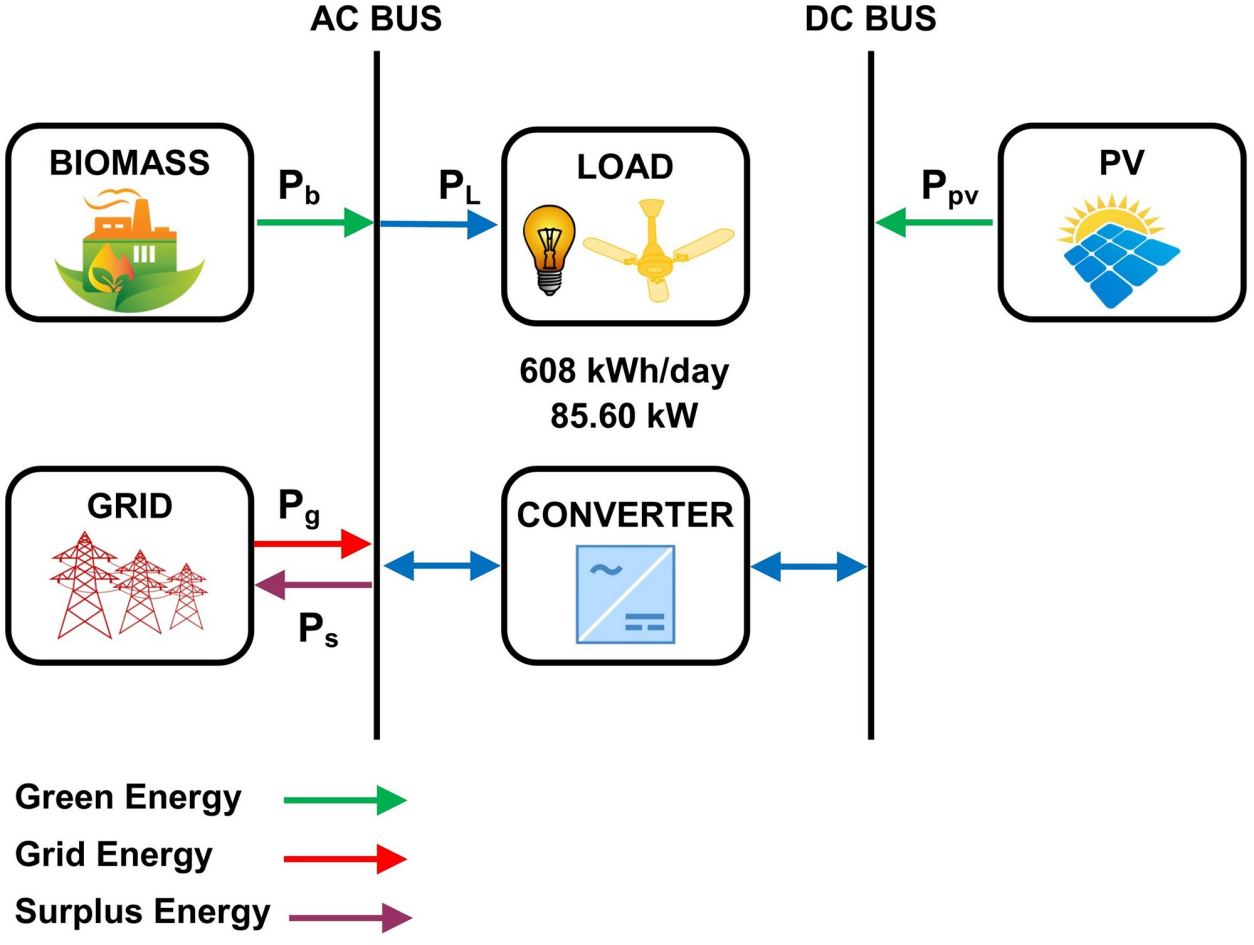
The hybrid energy model-C for the prosumer community.

PV modules, bi-directional converter and biomass-based generator are used as a system component in this HEM. HOMER simulates 64 different configurations for a search space range which is based on components’ size. Among these configurations, the optimal size of PV modules, converters and generator is 200 kW, 150 kW, and 50 kW, respectively. The capacity factor of PV modules and converters is 20% and 25%, respectively. The conversion losses of the system converter are 17296 kWh/year for 4386 hours of operation in a year. The electrical output efficiency is 25.9% for 8760 hours of operation. Fig 6 shows the hybrid energy model-C for the prosumer community.

Table 8 presents the simulation results for HEM-C. The energy contribution of photovoltaic modules, biomass-based system, and grid is 44.2%, 55.4%, and 0.374%, respectively. The community sell 547149 kWh/year of surplus energy with the grid at the rate of 0.08 USD/kWh. The number of carbon emissions that are released into the surroundings due to grid energy contribution is 1628 kgs/year. The net present cost is 8598 USD while the per unit cost of energy is 0.001 USD/kWh which is charged by the prosumer community. The capital cost, replacement cost, and operation and maintenance cost are 309000 USD, 146622 USD, and -439741 USD, respectively. The community generates revenue of 43771 USD/year by sharing surplus power with grid.

**Table 8:** Simulation results for model-C.

Component	Generation (kWh/year)	Grid sales (kWh/year)	CO_2_ emissions (kgs/year)	NPC (USD)	COE (USD/kWh)
PV	349646 (44.2%)	547149	1628	8598	0.001
Biomass	438000 (55.4%)				
Grid supply	2960 (0.374%)				
Total	790606 (100%)				

### 5.4 Comparative analysis of all hybrid energy models

**Fig 7:**
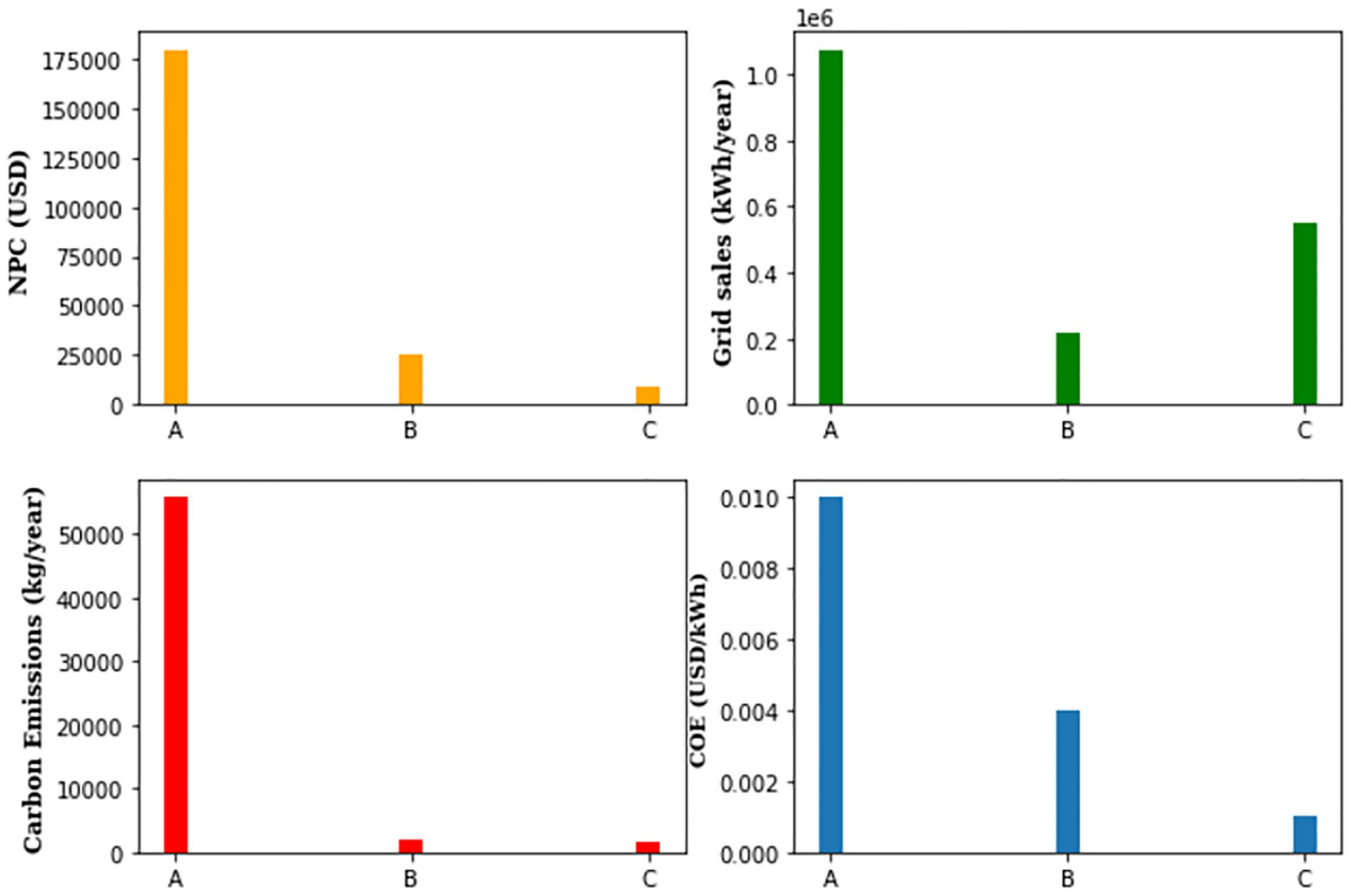
The comparative analysis of possible designs.

Fig 7 presents the comparative analysis in terms of net present cost, cost of energy, number of carbon emissions, and the surplus energy contribution to the grid for three different proposed hybrid energy models.

It is clear from Fig 7 that the NPC of HEM-A is more than 175000 USD which is high as compared to other proposed designs. Similarly, the per unit cost of energy and the number of carbon emissions is 0.01 USD/kWh and 55893 kgs/year for HEM-A. Regardless of high grid sales it cannot be considered an optimal design. It is also evident from Fig 7 that HEM-C is a more economical and ecosystem-friendly design in terms of NPC, COE, and the number of carbon emissions, respectively. The grid sales of HEM-C are 547149 kWh/year, and these are intermediate among the proposed designs. The HEM-B is neither economical nor ecosystem-friendly design when compared with HEM-C, however it is better than HEM-A.

### 5.5 The PV lifecycle-based sensitivity analysis

Photovoltaic modules lifetime-based sensitivity analysis is also performed to estimate the impact on economic feasibility of the optimal model-C. Table 9 presents the sensitivity analysis for net present cost, cost of energy and the optimal capacity. It is clear from table that with the decrease in module lifetime, the NPC rise. The NPC is 26495 USD for 20 years while it is 8598 USD for 25 years. The COE (USD/kWh) is inversely related with PV module lifetime. The COE is 0.003 USD/kWh for 20 years while 0.001 USD/kWh for 25 years. Moreover, the optimal capacity increase with the increase in module lifetime i.e., 150 kW for 20 years while 200 kW for 25 years.

**Table 9:** PV lifecycle-based sensitivity analysis.

Hybrid energy model	Lifetime (Year)	NPC (USD)	Optimal capacity (kW)	COE (USD/kWh)
C	20	26495	150	0.003
	25	8598	200	0.001

## 6. Conclusions

In this paper, the onsite survey data is simulated and optimized by using HOMER software to propose an economic and ecosystem-friendly hybrid energy model for a grid-connected prosumer community of 147 houses in the district Kotli, AJK. The HOMER proposed three hybrid energy models (HEM)-based on solar potential, available biomass sources, and grid supply. Among these models, HEM-C is an optimal proposed design to meet the energy demand of the targeted community based on the following grounds:

- The per unit cost of energy is reduced from 0.1 to 0.001 USD/kWh.
- The amount of carbon emissions is reduced from 122056 to 1628 kgs/year.
- A revenue of 43,771 USD/year is generated by sharing surplus energy with the grid.
- The renewable energy contribution of the proposed model is 99.6% while the fossil fuels-based grid energy contribution is only 0.374%.

The PV lifetime-based sensitivity analysis is performed which shows that NPC and COE are inversely proportional with modules’ life-span. It may be concluded from the results that the demand-supply gap can be reduced by the active participation of end users at a low per unit cost of energy in modern distribution systems. In addition, the renewable energy contribution will result in a sustainable power supply with less carbon emissions. However, the limitations of this research work for the implementation of the proposed model are: 1) the high cost of PV-modules, 2) fair inter and intra-energy trading models are not introduced yet to develop trust for increasing users’ participation, 3) the low rated current carrying capacity of distributors, 4) bi-directional power/information flow infrastructure is not installed, and 5) less environmental concern by people.

## Supporting information

S1 Dataset (RAR)
